# Paclitaxel Potentiates the Anticancer Effect of Cetuximab by Enhancing Antibody-Dependent Cellular Cytotoxicity on Oral Squamous Cell Carcinoma Cells In Vitro

**DOI:** 10.3390/ijms21176292

**Published:** 2020-08-31

**Authors:** Yuta Sawatani, Yuske Komiyama, Koh-ichi Nakashiro, Daisuke Uchida, Chonji Fukumoto, Michiko Shimura, Tomonori Hasegawa, Ryouta Kamimura, Masayo Hitomi-Koide, Toshiki Hyodo, Hitoshi Kawamata

**Affiliations:** 1 Department of Oral and Maxillofacial Surgery, Dokkyo Medical University School of Medicine, 880 Kita-kobayashi, Mibu, Shimo-tsuga, Tochigi 321-0293, Japan; sawayu@dokkyomed.ac.jp (Y.S.); y-komi@dokkyomed.ac.jp (Y.K.); chonji-f@dokkyomed.ac.jp (C.F.); smichiko@dokkyomed.ac.jp (M.S.); hase-t@dokkyomed.ac.jp (T.H.); kmry28@dokkyomed.ac.jp (R.K.); m-koide@dokkyomed.ac.jp (M.H.-K.); hyodo14@dokkyomed.ac.jp (T.H.); 2 Section of Dentistry and Oral and Maxillofacial Surgery, Sano Kosei General Hospital, 1728 Horigomecho, Sano, Tochigi 327-8511, Japan; 3 Department of Oral and Maxillofacial Surgery, Ehime University Graduate School of Medicine, Shitsukawa, Toon, Ehime 791-0295, Japan; nakako@m.ehime-u.ac.jp (K.-i.N.); udai@m.ehime-u.ac.jp (D.U.)

**Keywords:** antibody-dependent cellular cytotoxicity (ADCC), cetuximab, paclitaxel, synergistic effects, oral squamous cell carcinoma (OSCC), epidermal growth factor receptor (EGFR)

## Abstract

Administration of cetuximab (C-mab) in combination with paclitaxel (PTX) has been used for patients with head and neck squamous cell carcinoma (SCC) clinically. In this study, we attempted to clarify the molecular mechanisms of the enhancing anticancer effect of C-mab combined with PTX on oral SCC cells in vitro. We used two oral SCC cells (HSC4, OSC19) and A431 cells. PTX alone inhibited cell growth in all cells in a concentration-dependent manner. C-mab alone inhibited the growth of A431 and OSC19 cells at low concentrations, but inhibited the growth of HSC4 cells very weakly, even at high concentrations. A combined effect of the two drugs was moderate on A431 cells, but slight on HSC4 and OSC19 cells. A low concentration of PTX enhanced the antibody-dependent cellular cytotoxicity (ADCC) induced by C-mab in all of the cells tested. PTX slightly enhanced the anticancer effect of C-mab in this ADCC model on A431 and HSC4 cells, and markedly enhanced the anticancer effect of C-mab on OSC19 cells. These results indicated that PTX potentiated the anticancer effect of C-mab through enhancing the ADCC in oral SCC cells.

## 1. Introduction

The number of patients with head and neck cancer is reported to be the seventh most common cancer in the world [1]. Over 20,000 patients with head and neck cancer were treated in Japan in 2015 [2]. Oral cancer is classified as one of the head and neck cancer and includes the tumors developing in the buccal mucosa, maxillary gingiva, mandibular gingiva, hard palate, tongue, floor of the mouth, and lips [3]. Since the oral mucosa is covered by squamous epithelium, most oral cancers are histologically diagnosed as squamous cell carcinomas, but salivary gland cancer, malignant lymphoma, malignant melanoma, and mesenchymal tumors may also develop [3]. In the treatment of oral squamous cell carcinoma (SCC), surgery is mainly performed in combination with chemotherapy (including molecular targeted treatment), treatment with immune checkpoint inhibitors, and radiation therapy. With these treatments, overall survival (OS) and progression-free survival (PFS) in patients with oral SCC have improved considerably. In the department of Oral and Maxillofacial Surgery, Dokkyo Medical University School of Medicine, favorable therapeutic results have been obtained over the fourteen-year period from 2005 to 2018, as shown by the five-year OS rates of 87.5% for Stage I, 88.8% for Stage II, 80.9% for Stage III, and 62.5% for Stage IV, respectively [4]. However, some patients have shown local recurrence, cervical lymph node metastasis, and distal metastasis after the initial treatments, and it is difficult to achieve a complete cure for such patients. In the patients with local recurrence, lymph node metastasis, or distal metastasis, we hypothesized that dormant cancer cells that could not be detected before or during the initial treatment might rapidly or slowly grow after the primary treatment and develop into an obvious tumor. Therefore, we provide the additional treatment with a conventional anticancer drug, molecular targeted drug, or immunotherapy within three to six months after complete elimination of a visible tumor by surgery to attack the invisible dormant cancer cells and prevent local recurrence/metastasis [5].

Regarding chemotherapy for oral cancer in the guidelines for head and neck cancer of the National Comprehensive Cancer Network (NCCN) [6], it is suggested that after surgery for an advanced oral SCC, platinum-based chemotherapy is used as the first line. Concerning the general condition of patients with advanced oral SCC, it is often challenging to fit the platinum-based chemotherapy without changes. In many cases, they are exacerbated by malnourishment as well as comorbidities of lung, liver, and kidney [7]. Because of these matters, the guideline suggests concerning to personalize the treatment. In the “Head and Neck Cancer Drug Therapy Guidance” of the Japanese Society of Medical Oncology, it is suggested that “Administration of cetuximab (C-mab) in combination with paclitaxel (PTX) has been used for patients with head and neck cancer clinically. This combination is reported to be effective but with lower cytotoxicity compared to platinum-based chemotherapy” [8,9]. We also provide concomitant administration of C-mab and PTX in actual clinical practices and believe that this is an effective treatment with a high tolerability. However, the mutual enhancing mechanism of these drugs is unclear. We hypothesize that the essential antitumor effect of C-mab might be an induction of antibody-dependent cellular cytotoxicity (ADCC) [10], and that the cytotoxic effect of paclitaxel might enhance the ADCC induced by C-mab. In this study, we attempt to clarify the molecular mechanisms of the enhancing anticancer effect of C-mab combined with PTX on oral SCC cells in vitro.

## 2. Results

### 2.1. Effect of PTX and/or C-mab on the Growth of the Cells

We examined the effects of PTX (0.3–30,000 nM) and C-mab (0.01–1000 µg/mL) on the growth of the cells. PTX inhibited cell growth in all cells in a dose-dependent manner. C-mab inhibited the growth of A431 and OSC19 cells at low concentrations, and slightly inhibited the growth of HSC4, even at high concentrations. The 50% inhibitory concentration (IC50) of PTX on A431 cells was 60.6 nM, IC50 on HSC4 cells was 3448.1 nM, and IC50 on OSC19 cells was 401.9 nM. The IC50 of C-mab on A431 cells was 1.2 mg/mL, IC50 on HSC4 cells was 436.8 mg/mL, and IC50 on OSC19 cells was 2.1 mg/mL.

**Figure 1:**
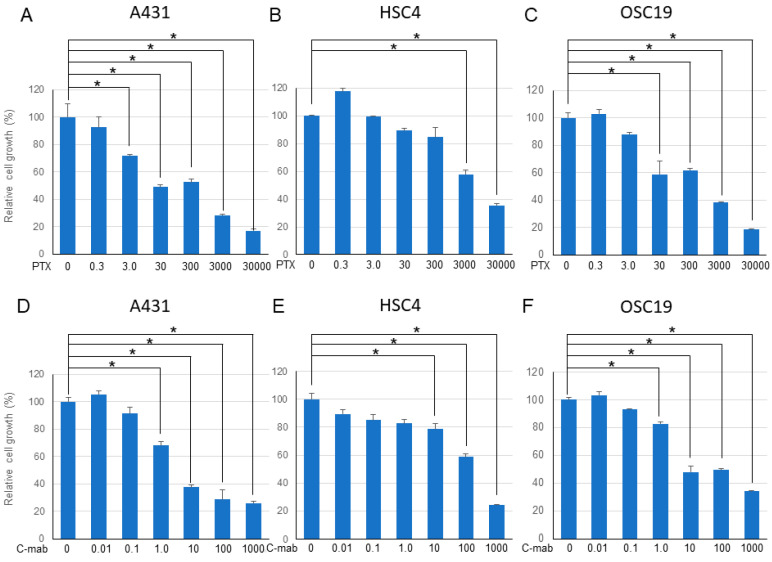
48 h after treatment. Relative cell growth with PTX treatment of each cell lines (**A**–**C**), or with C-mab treatment (**D**–**F**) are shown. Statistical analysis was performed by one-way ANOVA with Tukey’s multiple comparison test as a post hoc analysis. *: *p* < 0.05. The values shown are the mean of three determinations; bars: standard error of the mean. The data shown is a representative from three independent experiments with similar results.

**Figure 2:**
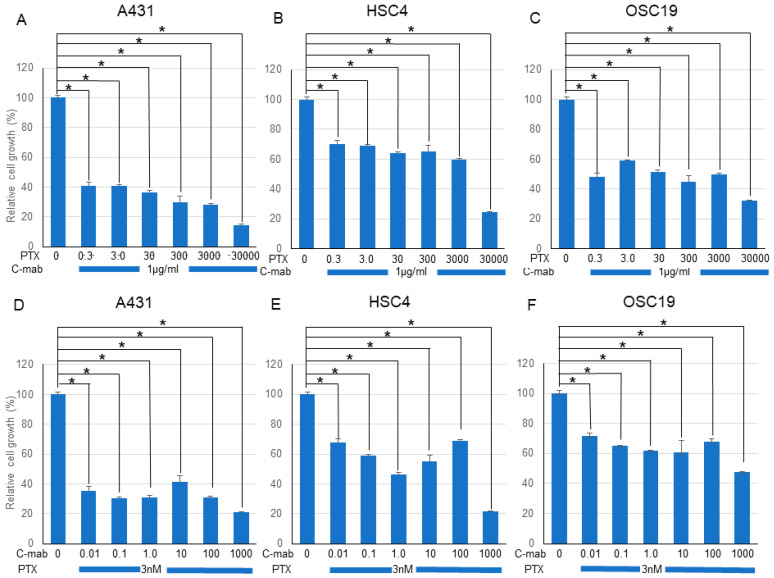
Effect of PTX and C-mab combinatory treatment to A431, HSC4, and OSC19 cell lines. Relative cell growth with PTX and C-mab combinatory treatment of each cell lines are shown. (**A**–**C**) C-mab concentration is fixed to 1.0 µg/mL and PTX conditions are adjusted from 0.3 to 30,000 nM. (**D**–**F**) PTX concentration is fixed to 3.0 nM and C-mab conditions are adjusted from 0.1 to 1000 µg/mL. Statistical analysis was performed by one-way ANOVA with Tukey’s multiple comparison test as a post hoc analysis. *: *p* < 0.05. The values shown are the mean of three determinations; bars: standard deviation. The data shown is a representative from three independent experiments with similar results.

Next, we examined the effects of a combination of PTX and C-mab on the growth of each cell line. PTX (Figure 1A–C) or C-mab (Figure 1D–F) alone inhibited cell growth dose-dependently during the 48 h after treatment in each cell line. The inhibitory effect of PTX on A431 at >3.0 nM, on HSC4 at >3000 nM, or on OSC19 at >30 nM was statistically significant (*p* < 0.05). The inhibitory effect of C-mab on A431 at >1.0 μg/mL, on HSC4 at >10 μg/mL, or on OSC19 at >1.0 μg/mL was statistically significant (*p* < 0.05). Furthermore, when the concentration of C-mab was fixed at 1.0 μg/mL and the concentration of PTX changed, combined effects were confirmed in all cells (Figure 2A–C). The combined inhibitory effect of PTX with 1 µg/mL C-mab on A431 at >0.3 nM, on HSC4 at >0.3 nM, or on OSC19 at >0.3 nM was statistically significant. Vice versa, when the concentration of PTX was fixed at 3.0 nM and the concentration of C-mab changed, combined effects were observed for A431, HSC4, and OSC19 (Figure 2D–F).The inhibitory effect of C-mab with PTX on A431 at >0.01 μg/mL, on HSC4 at >0.01 μg/mL, or on OSC19 at >0.01 μg/mL was statistically significant (*p* < 0.05).

We performed the Chou–Talalay method to assess the effect of the drug combination. The combination of PTX and C-mab synergistically inhibited the growth of the cells tested at most of the concentrations. The combination index (CI) for PTX (3.0 nM) and C-mab (1.0 µg/mL) was 0.01316 in A431, 0.02140 in HSC4, and 0.01740 in OSC19.

### 2.2. ADCC Assay

**Figure 3:**
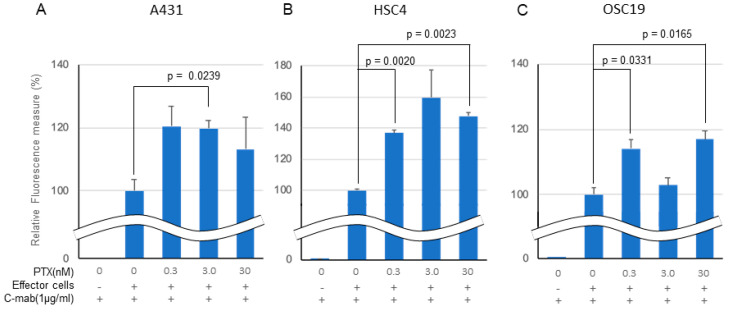
Effect of PTX on antibody-dependent cell-mediated cytotoxicity. Relative fluorescence to C-mab plus effector cells with gradient PTX concentration are shown. (**A**) A431, (**B**) HSC4, (**C**) OSC19. C-mab concentration was fixed to 1.0 µg/mL and 75,000 effector cells was added to induce ADCC. Double waved line indicates the contraction. Statistical analysis was performed by one-way ANOVA with Student’s *t*-test as a post hoc analysis, *p* values are indicated. The values shown are the mean of three determinations; bars: standard error of the mean. The data shown is a representative from three independent experiments with similar results.

In an in vitro ADCC model, C-mab (1.0 µg/mL) exhibited ADCC activity in all of the cell lines tested when Jurkat cells were used as effector cells (Figure 3A–C). Low concentration PTX enhanced the ADCC activity by C-mab in all of the cells tested. The enhancing effect of PTX on ADCC activity in the ADCC model reached significance in the A431 cells (3.0 nM PTX: *p* = 0.0239), in the HSC4 cells (0.3 nM PTX: *p* = 0.0020, 30 nM PTX: *p* = 0.0023), and in the OSC19 cells (0.3 nM PTX: *p* = 0.0331, 30 nM PTX: *p* = 0.0165), respectively, although it did not reach a significant level in the A431 cells (0.3 nM PTX: *p* = 0.0973, 30 nM PTX: *p* = 0.4037), in the HSC4 cells (3.0 nM PTX: *p* = 0.1095), and in the OSC19 cells (3.0 nM PTX: *p* = 0.4631). We performed three separate experiments and obtained similar results, and present a representative finding. We also tested rituximab, the anti CD20 antibody, as a negative control for the in vitro ADCC assay (data not shown).

### 2.3. Cell Killing Activity by ADCC

**Figure 4:**
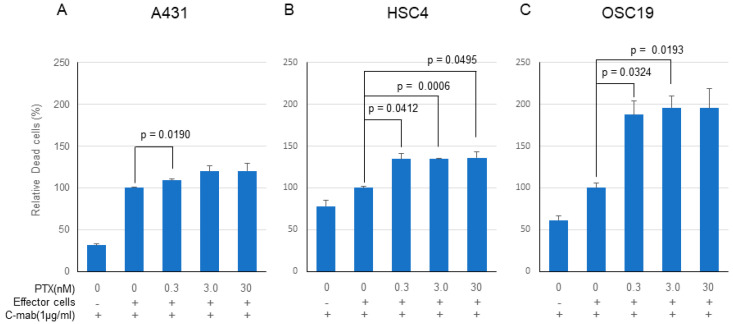
Cell-killing activity of PTX and C-mab combination treatment with ADCC induction. Relative dead cells to C-mab plus effector cells with gradient PTX concentration are shown. (**A**) A431, (**B**) HSC4, (**C**) OSC19. Statistical analysis was performed by one-way ANOVA with Student’s *t*-test as a post hoc analysis, statistical significance is indicated. The values shown are the mean of three determinations; bars: standard error of the mean. The data shown is a representative from three independent experiments with similar results.

PTX significantly enhanced the anticancer effect of C-mab in the ADCC model in all of the cells tested, although the enhancing effects did not reach the significant level on some cells (Figure 4A–C). Enhancing cell killing effect of PTX on ADCC by C-mab was approximately 120% in the A431 cells (0.3 nM PTX: *p* = 0.0191, 3.0nM PTX: *p* = 0.1450, 30 nM PTX: *p* = 0.2256), approximately 140% in HSC4 cells (0.3 nM PTX: *p* = 0.0412, 3.0 nM PTX: *p* = 0.0006, 30 nM PTX: *p* = 0.0495), and approximately 200% on OSC19 cells (0.3 nM PTX: *p* = 0.0324, 3.0 nM PTX: *p* = 0.0193, 30 nM PTX: *p* = 0.0690). Since the high concentration of PTX (300 nM or higher) kills the effector cells, cell-killing experiments by ADCC were performed on the concentration of PTX at lower than 30 nM.

### 2.4. NGS Analysis for Driver Genes on Oral SCC

p53 gene was mutated in all of the cell lines (HSC4: R248Q, OSC19: K164 *, A431: R273H), Notch1 was mutated in A431 (P1377S), and PIK3CA was mutated in HSC4 (E545K). None of the other genes examined (KRAS, HRAS, BRAF, Akt, EGFR, PTEN, CDKN2A, FBXW7) were mutated in the coding region in any of the cells.

### 2.5. Microarray Analysis

The expression of the molecules related to EGFR signaling were picked up from the microarray data and compared among the cells (Figure S1A–C). Although the expression of EGFR, KRAS, HRAS, NRAS, BRAF, MEK, ERK, Akt, PI3K, etc. varied depending on the cells, none of the molecules showed any signs of loss of expression or significantly enhanced expression. Thus, we believed that EGFR signaling was functional in all cells.

### 2.6. Effect of PTX on the Expression of EGFR in All of the Cells

**Figure 5:**
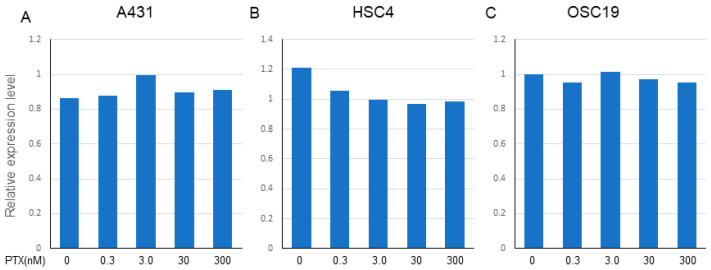
Effect of PTX on the expression of EGFR in A431, HSC4, and OSC19. The expression of EGFR on each cell line was determined by TaqMan PCR after treatment with gradient conditions from 0.3 to 300 nM of PTX. (**A**) A431, (**B**) HSC4, (**C**) OSC19. Relative expression level of EGFR (C_t_ value) to the beta-actin as a housekeeping gene is expressed. The data shown is a representative PCR result from two independent experiments with similar results.

PTX (0.3–300 nM) did not affect the expression of EGFR in A431 and OSC19 cells, but marginally decreased the expression of EGFR in HSC4 cells (Figure 5A–C). We conducted two independent treatments and obtained similar PCR results.

## 3. Discussion

In this experiment, we examined the effect of PTX (0.3–30,000 nM) and C-mab (0.01–1000 µg/mL) on cell growth. PTX alone inhibited cell growth in all cells in a concentration-dependent manner. C-mab alone inhibited the growth of A431 and OSC19 cells at low concentrations, but inhibited the growth of HSC4 cells very weakly, even at high concentrations. A synergistic effect of the two drugs was observed on all of the cells tested. Subsequently, we examined the effects of PTX on the ADCC activity of C-mab. C-mab (1 µg/mL) exhibited ADCC activity in all of the cells tested. Low concentration PTX enhanced the ADCC activity induced by C-mab in all of the cells tested. PTX slightly enhanced the anticancer effect of C-mab in this ADCC model on A431 and HSC4 cells, and markedly enhanced the anticancer effect of C-mab on OSC19 cells. These results indicated that PTX potentiated the anticancer effect of C-mab through enhancing the ADCC in oral SCC cells.

The expression of EGFR in patients with oral cancer is elevated compared with that in patients with other cancer, such as colorectal cancer or bladder cancer [11]. However, as observed in the EGFR expression in the A431 cells, it was confirmed that the oral cancer cells express not only full-length EGFR but also EGFR lacking the intracellular domain [12,13]. Therefore, EGFR works as both a signal transducer, as well as a so-called tumor marker. In addition, proliferation of cancers including oral cancer is not necessarily promoted by exogenous stimuli, such as EGF and TGF-α, and sometimes, such exogenous ligands may inhibit the growth of the cells [14,15,16]. Therefore, it is difficult to consider that antitumor effects would be obtained by blocking EGFR signaling alone in patients with oral cancer. It has been reported that panitumumab, a complete human IgG2 antibody, would be effective for colorectal cancer, but not for head and neck cancers, including oral cancer [17]. On the other hand, since C-mab is a chimeric antibody and its subtype is IgG1, C-mab is considered to strongly cause ADCC and other immunological reactions [18]. Therefore, we believe that the main action of C-mab in patients with oral cancer may be an immunological effect, such as ADCC, rather than signaling blockade.

Since it is known that EGFR expression is high in most cells in patients with oral cancer, and only a few mutation would be observed in BRAF and RAS [19], which are key intracellular signaling molecules, it is not necessary to confirm expression of EGFR and the absence of BRAF and RAS mutations to use C-mab, unlike the cases of colorectal cancer. In addition, it is known that some oral cancers express EGFRvIII, a truncated molecule lacking a part of extracellular domain, which shows ligand-independent signaling [20]. Therefore, some researchers believe that EGFRvIII is a marker of C-mab resistance [21]. However, it is also known that C-mab also binds to EGFRvIII, although signaling cannot be blocked, but ADCC can be induced [22]. In the oral cancer cells (HSC4, OSC19) that were used in this study, high expression of EGFR was confirmed, but no mutations of BRAF, HRAS, KRAS, and NRAS were observed. Furthermore, although mutation and loss of expression of molecules related to the classic MAPK and mTOR-AKT pathways were not observed, the direct action of C-mab was limited in vitro. Although the cell-killing effect of PTX was confirmed, the effect was limited at a concentration equivalent to clinical concentration. All of the cells used in this experiment had a mutation in the p53 gene DNA binding domain, but the response to the drugs were not related to the type (missense or nonsense) and site of p53 mutation. Furthermore, expression status of the upstream and downstream molecules of EGFR could not explain the different response of the cells to C-mab. Further experiments might be necessary to explain the relation between different response of the cells to drugs and the genetic and expression status of the related genes.

In this study, we used a model to measure in vitro ADCC activity to directly verify our hypotheses. As expected, C-mab induced ADCC in oral cancer cells with expression of EGFR, thus showing the cell-killing effect. Furthermore, PTX potentiated the ADCC effect of C-mab and cell-killing effects in all cell lines. The ADCC promoting effect and the cell-killing effects were significant in all cell lines. However, no clear correlation was observed between the ADCC promoting effect and the cell-killing effect. When we examined the difference in mutation of the driver gene and expression of molecules related to EGFR signaling, cell cycle, and the induction of apoptosis, no differences were confirmed that would clearly explain the difference in these responses.

To our knowledge, this is the first report that PTX potentiates the anticancer effect of C-mab by enhancing ADCC on oral SCC cells in vitro. PTX is known to inhibit microtubule polymerization and induce cell death by mitotic inhibition. However, at present, the mechanism of the action of PTX to increase ADCC remains unclear. Since C-mab can be considered as an immunotherapy drug, PTX might exhibit some effects to increase immunogenicity of the cells. Since almost no effects of PTX on the expression of EGFR were observed, the involvement of EGFR was considered to be low. Hyperploidy was induced when PTX inhibited mitosis, causing so-called mitotic catastrophe, which may lead to increased immunological reactions [23]. As seen by the significant effects of immune checkpoint inhibitors on cancer cells with mutations of the gene for mismatch repair enzymes [24,25], and cells with high tumor mutation burden and expression of neoantigen [26], linking of the immune reaction may be induced in mitotic catastrophe. We are currently investigating the underlying mechanisms for enhancing effect of PTX on the ADCC induced by C-mab on oral SCC cells.

## 4. Materials and Method

### 4.1. Cell Lines and Cell Culture

We used two oral SCC cell lines (EGFR high: HSC4, EGFR low: OSC19) and A431 cells. A431 cells were established from a vulval SCC and are well known to overexpress EFGR. These cell lines were purchased from the Japanese Collection of Research Bioresources Cell Bank (Tokyo, Japan). These cell lines were cultured in 100 mm culture dishes and maintained with Dulbecco’s modified Eagle’s medium (DMEM; SIGMA, Tokyo, Japan) supplemented with 10% fetal bovine serum (FBS; gibco, Tokyo, Japan), and 5.0% antibiotic-antimycotic solution (FUJIFILM, Osaka, Japan). In all experiments, cells were incubated at 37.0 °C in a humidified atmosphere containing 5.0% CO_2_. To confirm the origin and identity of the cells used in this experiment, we performed a short tandem repeat (STR) analysis (Tables S1 and S2).

### 4.2. Effect of PTX and/or C-mab on the Cell Growth

To examine the effects of PTX and C-mab on each cell line, PTX was added at doses of 0, 0.3, 3.0, 30, 300, 3000, and 30,000 nM, while C-mab was added at doses of 0, 0.1, 1.0, 10, 100, and 1000 μg/mL. Next, to examine whether PTX would increase the effects of C-mab at a fixed dose, the dose of C-mab was fixed at 1.0 μg/mL, and PTX was added at various doses (0, 0.3, 3.0, 30, 300, 3000, and 30,000 nM). Furthermore, to examine whether C-mab would increase the effects of PTX at a fixed dose, PTX was concomitantly added at a dose of 3.0 nM and C-mab was added at various doses (0, 0.1, 1.0, 10, 100, and 1000 μg/mL). The number of cells in any examination was analyzed using the Cell Counting Kit-8 (CCK-8; Dojindo, Tokyo, Japan). Statistical analysis was performed by one-way ANOVA with Tukey’s multiple comparison test as a post hoc analysis. *: *p* < 0.05.

### 4.3. ADCC Assay

ADCC activity was examined using the ADCC Reporter Bioassay, Complete Kit (Raji) purchased from Promega Japan (Tokyo, Japan). Target cells (A431, HSC4, OSC19) were seeded at 5000/well, and after 24 h, C-mab (1 μg/mL) and PTX (0.3–300 nM) were added. After an additional 42 h, the effector cells, Jurkat cells (human T cell leukemic cells), were inoculated at 75,000/well. Then luciferase activity was measured using a luminometer 6 h after inoculation. The Jurkat cells used in our study expresses FcgR on cell surface, and when they bind together with the Fc region of antibody binding to the antigen of the target cell, its signal activates the NFAT (nuclear factor of activated T-cells) pathway. Since the cells express the reporter luciferase gene, under control of a NFAT responsive element, ADCC activity can be evaluated by measuring luciferase activity with Infinite 200 PRO (TECAN, Mannedorf, Switzerland). Statistical analysis was performed by one-way ANOVA with Student’s *t*-test as a post hoc analysis; *p* values are indicated.

### 4.4. Killed Cells Evaluated by ADCC Activity

To evaluate cell killing of the target cells by ADCC, killed cells were stained with propidium iodide and counted. Since highly concentrated PTX killed the effector cells, Jurkat cells (data not shown), the cells killed by ADCC when PTX was added at concentrations of lower than 30 nM, were stained with propidium iodide counted using an auto-cell counter (BZ-H3C: KEYENCE, Osaka, Japan). Statistical analysis was performed by one-way ANOVA with Student’s *t*-test as a post hoc analysis; statistical significance is indicated.

### 4.5. Microarray

Total RNA was extracted by lysing the tissues using ISOGEN (Nippon Gene: Tokyo, Japan), according to the manufacturer’s instructions, and homogenizing them in 0.5 mL of ISOGEN using a Tissue Lyser (Qiagen, Valencia, CA, USA). The integrity of the RNA was confirmed using an Agilent 2100 Bioanalyzer (Agilent Technologies, Santa Clara, CA, USA). An Applied Biosystems Chemiluminescent RT-IVT Labeling Kit (Life Technologies, Carlsbad, CA, USA) was used to convert total RNA to digoxigenin (DIG)-labeled cRNA. Double-stranded cDNA was generated from 1 µg of total RNA, transcribed using DIG-labeled nucleotides (Roche Diagnostics, Basel, Switzerland), fragmented, and hybridized to a Human Genome Survey Array containing 32,878 probes (Life Technologies, Carlsbad, CA, USA) according to the manufacturer’s instructions. After washing each array, the signal was developed using a chemiluminescent detection kit (Life Technologies, Carlsbad, CA, USA). Processed arrays were scanned with a 1700 chemiluminescent microarray analyzer (Life Technologies, Carlsbad, CA, USA) and the results were then analyzed using GeneSpring GX ver. 13.0 (Agilent Technologies, Santa Clara, CA, USA).

### 4.6. Next Generation Sequencing

NGS analysis was performed using an in-house panel. Sequences of whole exons which covered the open reading frame for of EGFR, HRAS, KRAS, BRAF, PIK3CA, Akt, TP53, PTEN, NOTCH1, and CDKN2A were analyzed. First, 646 ng of genomic DNA were fractionated into pieces of several hundred base pairs, and the library was constructed using TruSeq Custom Amplicon Low Input (Illumina, Santiago, CA, USA) and individual custom oligonucleotides for EGFR, HRAS, KRAS, BRAF, PIK3CA, Akt, TP53, PTEN, NOTCH1, and CDKN2A. Using TruSeq Custom Amplicon Index Kit (Illumina, Santiago, CA, USA), an identification sequence and two types of adapter were added. The concentration of the library was adjusted for each specimen, and sequencing was performed with a next-generation sequencer (MiSeq, Illumina, Santiago, CA, USA) after mixing of the library with a MiSeq Reagent Kit v2 (300 Cycles) (Illumina, Santiago, CA, USA). Data were uploaded to Illumina VariantStudio ver.3.0 (Illumina, Santiago, CA, USA) to extract mutations.

### 4.7. Examination of the Effect of PTX on EGFR mRNA Expression in Each Cell Line by TaqMan PCR

After each cell line was cultured in six well plates until subconfluency, PTX was added at doses of 0–300 nM for 48 h. Total RNA from the cells was extracted using a modified acid guanidinium–isothiocyanate–phenol–chloroform method with the ISOGEN RNA extraction mixture (Nippon Gene, Tokyo, Japan) according to the manufacturer’s recommendations. cDNA was synthesized using random primers (Takara Bio, Shiga, Japan) and M-MLV reverse transcriptase (Takara Bio, Shiga, Tokyo) from 5 µg of total RNA. Using TaqMan Universal Master MixII, we performed real time PCR for EGFR. β-actin was used an internal control.

## Supplementary Materials

Supplementary materials can be found at https://www.mdpi.com/1422-0067/21/17/6292/s1.

## Abbreviations

PTX	Paclitaxel
C-mab	Cetuximab
SCC	Squamous cell carcinoma
ADCC	Antibody-dependent cellular cytotoxicity
OSCC	Oral squamous cell carcinoma
EGFR	Epidermal growth factor receptor
OS	Overall survival
PFS	Progression-free survival
NCCN	National Comprehensive Cancer Network
IC50	50% inhibitory concentration
ANOVA	Analysis of variance
PCR	Polymerase chain reaction
EGF	Epidermal growth factor
KRAS	V-Ki-ras2 Kirsten rat sarcoma viral oncogene homolog
HRAS	V-Ha-ras Harvey rat sarcoma viral oncogene homolog
BRAF	V-raf murine sarcoma viral oncogene homolog
Akt	V-akt murine thymoma viral oncogene homolog
PTEN	Phosphatase and tensin homolog
CDKN2A	Cyclin-dependent kinase inhibitor 2A (melanoma, p16, inhibits CDK4)
FBXW7	F-box and WD repeat domain containing 7
