# Supplementary material for: Paclitaxel Potentiates the Anticancer Effect of Cetuximab by Enhancing Antibody-Dependent Cellular Cytotoxicity on Oral Squamous Cell Carcinoma Cells In Vitro
Source: Int J Mol Sci. 2020 Aug 31;21(17):6292. doi: 10.3390/ijms21176292 (PMC7503545; doi:10.3390/ijms21176292)
Supplement: Supplementary file 1 [file ijms-21-06292-s001.pdf]

Table S1. STR analysis.

A.

Sample Name	Marker	Allele1	Allele2
A431	AMEL	X	
A431	CSF1PO	11	12
A431	D13S317	9	13
A431	D16S539	12	14
A431	D21S11	28	
A431	D5S818	12	13
A431	D7S820	10	
A431	TH01	9	
A431	TPOX	11	
A431	vWA	15	17

B.

Sample Name	Marker	Allele1	Allele2
HSC-4	AMEL	X	
HSC-4	CSF1PO	9	10
HSC-4	D13S317	8	13
HSC-4	D16S539	12	
HSC-4	D21S11	29	30
HSC-4	D5S818	8	11
HSC-4	D7S820	9	10
HSC-4	TH01	6	9
HSC-4	TPOX	11	
HSC-4	vWA	16	19

C.

Sample Name	Marker	Allele1	Allele2
OSC-19	AMEL	X	Y
OSC-19	CSF1PO	12	
OSC-19	D13S317	12	
OSC-19	D16S539	9	12
OSC-19	D21S11	21	
OSC-19	D5S818	10	13
OSC-19	D7S820	9	11
OSC-19	TH01	9	
OSC-19	TPOX	11	
OSC-19	vWA	14	

Table S2. Evaluation value.

	**A431**	**HSC4**	**OSC19**
A431	-	0.333	0.370
HSC4	-	-	0.276
OSC19	-	-	-

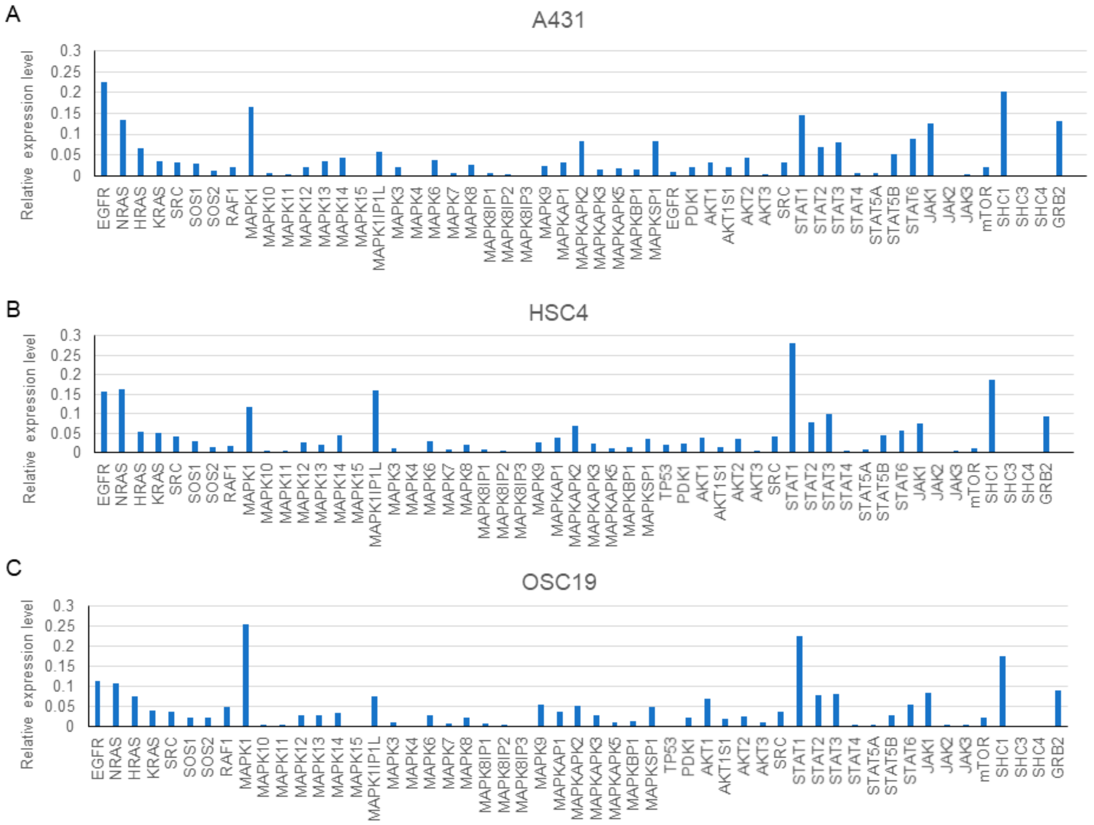

Figure S1A, S1B, S1C: Expression levels of various genes closely related to EGFR signaling analyzed with microarray. The expression levels of each genes closely related to EGFR signaling were indicated. Values were calculated the relative expression to the expression level of GAPDH as a house-keeping gene. (A) A431, (B) HSC4, (C) OSC19.
